# Quantum-based machine learning and AI models to generate force field parameters for drug-like small molecules

**DOI:** 10.3389/fmolb.2022.1002535

**Published:** 2022-10-11

**Authors:** Sathish Kumar Mudedla, Abdennour Braka, Sangwook Wu

**Affiliations:** ^1^ R&D Center, PharmCADD, Busan, South Korea; ^2^ Department of Physics, Pukyong National University, Busan, South Korea

**Keywords:** partial charge prediction, AI force field, atomtype prediction, protein ligand binding, free energy, atomic charges

## Abstract

Force fields for drug-like small molecules play an essential role in molecular dynamics simulations and binding free energy calculations. In particular, the accurate generation of partial charges on small molecules is critical to understanding the interactions between proteins and drug-like molecules. However, it is a time-consuming process. Thus, we generated a force field for small molecules and employed a machine learning (ML) model to rapidly predict partial charges on molecules in less than a minute of time. We performed density functional theory (DFT) calculation for 31770 small molecules that covered the chemical space of drug-like molecules. The partial charges for the atoms in a molecule were predicted using an ML model trained on DFT-based atomic charges. The predicted values were comparable to the charges obtained from DFT calculations. The ML model showed high accuracy in the prediction of atomic charges for external test data sets. We also developed neural network (NN) models to assign atom types, phase angles and periodicities. All the models performed with high accuracy on test data sets. Our code calculated all the descriptors that were needed for the prediction of force field parameters and produced topologies for small molecules by combining results from ML and NN models. To assess the accuracy of the predicted force field parameters, we calculated solvation free energies for small molecules, and the results were in close agreement with experimental free energies. The AI-generated force field was effective in the fast and accurate generation of partial charges and other force field parameters for small drug-like molecules.

## 1 Introduction

Molecular dynamics (MD) simulations play an important role in rational drug design, (Marco De, et al., 2016), which is useful in the analysis of dynamical interactions between a target protein and drug molecules (Allinger, 1977; Lifson, et al., 1979; Burkert and Allinger, 1982; Brooks, et al., 1983; Jorgensen and Tirado-Rives, 1988; Allinger, et al., 1989; Clark, et al., 1989; Mayo, et al., 1990; Momany and Rone, 1992; Rappé et al., 1992; Hwang, et al., 1994; Cornell, et al., 1995; Halgren, 1996; Wang, et al., 2000; Wang, et al., 2005). MD simulations are less accurate than first principles approaches. However, a well-parameterized force field can be used to produce results comparable to those of quantum mechanical (QM) calculations (Weiner, et al., 1984). The dynamical properties of proteins, and DNA and RNA molecules can be described by performing MD simulations using well-established traditional force fields such as AMBER, CHARMM, GROMOS and OPLS-AA (Jorgensen, et al., 1996; Mackerell, et al., 1998; Ponder and Case, 2003; Oostenbrink, et al., 2004; Zgarbova, et al., 2011; Bergonzo and Cheatham, 2015; Maier, et al., 2015; Vanommeslaeghe and MacKerell, 2015; Galindo-Murillo, et al., 2016; Tian, et al., 2019). The generation of parameters for fundamental units for biological macromolecules is sufficient to describe the properties of proteins, DNA and RNA. However, the force field for small organic molecules should cover a large chemical space because each drug-like molecule contains different chemical fragments.

In general, a force field consists of bonded and nonbonded parameters (Jorgensen, et al., 1996; Mackerell, et al., 1998; Ponder and Case, 2003; Oostenbrink, et al., 2004; Zgarbova, et al., 2011; Bergonzo and Cheatham, 2015; Maier, et al., 2015; Vanommeslaeghe and MacKerell, 2015; Galindo-Murillo, et al., 2016; Tian, et al., 2019). Nonbonded parameters are van der Waals and electrostatic atomic charges. In molecular simulations, electrostatics are calculated using atom-centered point charges with the aid of a simple Coulombic model. The electrostatic energy component is the dominant one in nonbonded interactions such as ligand binding to a receptor, therefore, the generation of qualitative atomic charges plays a key role in studying the binding of ligands to receptors using simulations (Honig and Nicholls, 1995). An atomic charge should include the influence of the corresponding atom and its bonded atoms. Additionally, the point charge must account for the electronic effects from nearby electron-donating or electron-withdrawing functional groups and formal charges in the molecule (Jakalian, et al., 2002). Hence, charge models should take into account all these effects.

To generate electrostatics for a molecule, it is necessary to perform QM calculations. Several software packages, such as antechamber (Wang, et al., 2006) and CGenff (Vanommeslaeghe, et al., 2010) generate force field parameters for small organic molecules using quantum mechanical calculations at different levels. Charge methods, including AM1-BCC, CGenFF, CM1A, CM3P and CM5, are used in conjunction with AMBER, CHARMM and OPLS force fields to generate force field parameters for drug-like molecules (Storer, et al., 1995; Jakalian, et al., 2000; Jakalian, et al., 2002; Thompson, et al., 2003; Marenich, et al., 2012). The charge methods CM1A (Storer, et al., 1995), CM3P (Thompson, et al., 2003) and AM1-BCC (Jakalian, et al., 2000; Jakalian, et al., 2002) and produce atomic charges by applying different empirical corrections to charges derived from semiempirical quantum methods such as AM1 and PM3. CM5 produces charges using Hirshfeld population analysis with the aid of density functional theory (DFT) methods (Marenich, et al., 2012). To consider the polarization effect by the environment, these methods increase the magnitude of charges by using scaling factors such as 1.14 for CM1A3 and 1.20 for CM5 (Udier-Blagovic, et al., 2004; Vilseck, et al., 2014). AM1-BCC utilizes bond-based incremental corrections to the charges obtained by Mulliken population analysis (Jakalian, et al., 2000). Bond charge corrections are parametrized by fitting to HF/6-31G* ESP of molecules in the training set (Jakalian, et al., 2000). These models have both pros and cons. For instance, AM1-BCC successfully describes electrostatics for nonpolar molecules such as saturated and aromatic hydrocarbons. However, it fails in the case of polar molecules such as pyridines, alkyl amines, alkyl and aryl halides, sulfides, and nitriles (Jakalian, et al., 2000; Jakalian, et al., 2002). The DFT-derived CM5 model suffers from a lack of a fixed scale factor to account for internal electron delocalization and external polarization effects (Marenich, et al., 2012). Recently, the 1.14*CM1A charge model with localized bond charge corrections showed high accuracy in reproducing experimental solvation free energies and heat of vaporization and densities with relatively small errors (Dodda, et al., 2017). In addition to AM1-BCC charge method, antechamber produces RESP charges using the ESP charges from user provided QM calculations for the molecule. CGenff program initially estimates ESP charges from the optimization calculations at MP2/6-31G* level which is computationally expansive to perform. Then it further optimizes the charges based on the QM data for the molecule which is interacting with water molecules in various orientations. Thus, the popular Antechamber and CGenff methods use ESP charges from different levels of theory and then introduces corrections to further improve the quality of charges. Despite the success in charge models, it is necessary to develop charge models which are optimized for efficiency and accuracy for small molecules to the accurate estimation of electrostatics in MD simulations.

Machine learning algorithms have been successfully applied to the generation of new scaffolds of small drug-like molecules (Lavecchia, 2015; Lipinski, et al., 2019; Patel, et al., 2020; Carracedo-Reboredo Jose et al., 2021), toxicity prediction (Wu and Wang, 2018), and omics pattern recognition (Stanke and Morgenstern, 2005). Machine learning algorithms have also been applied to predict partial charges and forces on atoms of small molecules in the field of quantum chemistry (Roman and Dominik, 2019; Pattnaik, et al., 2020). The calculated force on the atom in a molecule is used to perform *ab initio* MD simulations. The contribution of electrostatic interactions is prominent in force field-based MD simulations (Jorgensen, 2005). The atomic charges of molecules alter the interaction with water thus sensitive to condensed phase properties including free energies of hydration and heats of vaporization (Jorgensen and Tirado-Rives, 2005). The accurate estimation of electrostatic interactions between proteins and ligands is important in calculating binding free energies, which are useful for screening small molecules in computer-aided drug design (Jorgensen 2009). Despite the progress in the polarizable force fields, the point charge models are still essential owing to their low computational cost and accuracy (Swope, et al., 2010). Hence, in this study, we have developed machine learning and DFT charge-based artificial intelligence (AI) models to predict atomic charges and to generate force fields for small molecules in less than a minute of time.

## 2 Computational methods

### 2.1 Force field parameters

The potential energy is the sum of the nonbonded (van der Waals and electrostatic) and bonded (bonds, angles and dihedrals) interactions in a molecule. The general functional form of potential energy in force fields is as follows in eqn. 1. (Jorgensen, et al., 1996; Mackerell, et al., 1998; Ponder and Case, 2003; Oostenbrink, et al., 2004; Zgarbova, et al., 2011; Bergonzo and Cheatham, 2015; Maier, et al., 2015; Vanommeslaeghe and MacKerell, 2015; Galindo-Murillo, et al., 2016; Tian, et al., 2019).

$$V=\underset{bonds}{\sum }K_{b}{\left(r-r_{0}\right)}^{2}+\underset{angles}{\sum }K_{\theta }{\left(\theta -\theta_{0}\right)}^{2}+\underset{dihedrals}{\sum }K_{\phi }\left[1+\cos\left(n\phi -\gamma \right)\right]+\underset{i,j\;pairs}{\sum }\left(\frac{A_{ij}}{r_{ij}^{12}}-\frac{B_{ij}}{r_{ij}^{6}}\right)+\underset{i,j\;pairs}{\sum }\frac{q_{i}q_{j}}{\varepsilon r_{ij}}$$ (1)

where K_b_ = force constant of bond, K_θ_ = force constant of angle, K_ϕ_ = force constant of dihedral angle, r = bond length, r_0_ = equilibrium bond length, θ_0_ = equilibrium angle, θ = angle, ϕ = dihedral angle, ϕ_0_ = equilibrium dihedral angle, q_i_, q_j_ = partial charges, A_ij_, B_ij_ = well depth and r_ij_ = distance.

All the above-mentioned force field parameters are necessary to calculate the potential energy in MD simulations. In this study, we aimed to generate all these force field parameters except van der Waals potentials for drug molecules using machine learning tools. The existing van der Waals parameters for the atom types of organic molecules were developed with great care by matching the densities and enthalpies of vaporization (Cornell, et al., 1995; Jorgensen, et al., 1996). The van der Waals parameters also developed using QM methodologies and they were refined by fitting experimental properties including heat of vaporization, molecular volume and hydration free energy (Rupakheti et al., 2018) and it needs an extensive of work to achieve. Also, the small changes in van der Waals potentials cause significant changes in the properties of molecules in the solution (Rupakheti et al., 2018; Boulanger et al., 2021). Therefore, in this study, we have not focused on the development of new van der Waals parameters using machine learning algorithms.

### 2.2 Generation of the training data set

**FIGURE 1:**
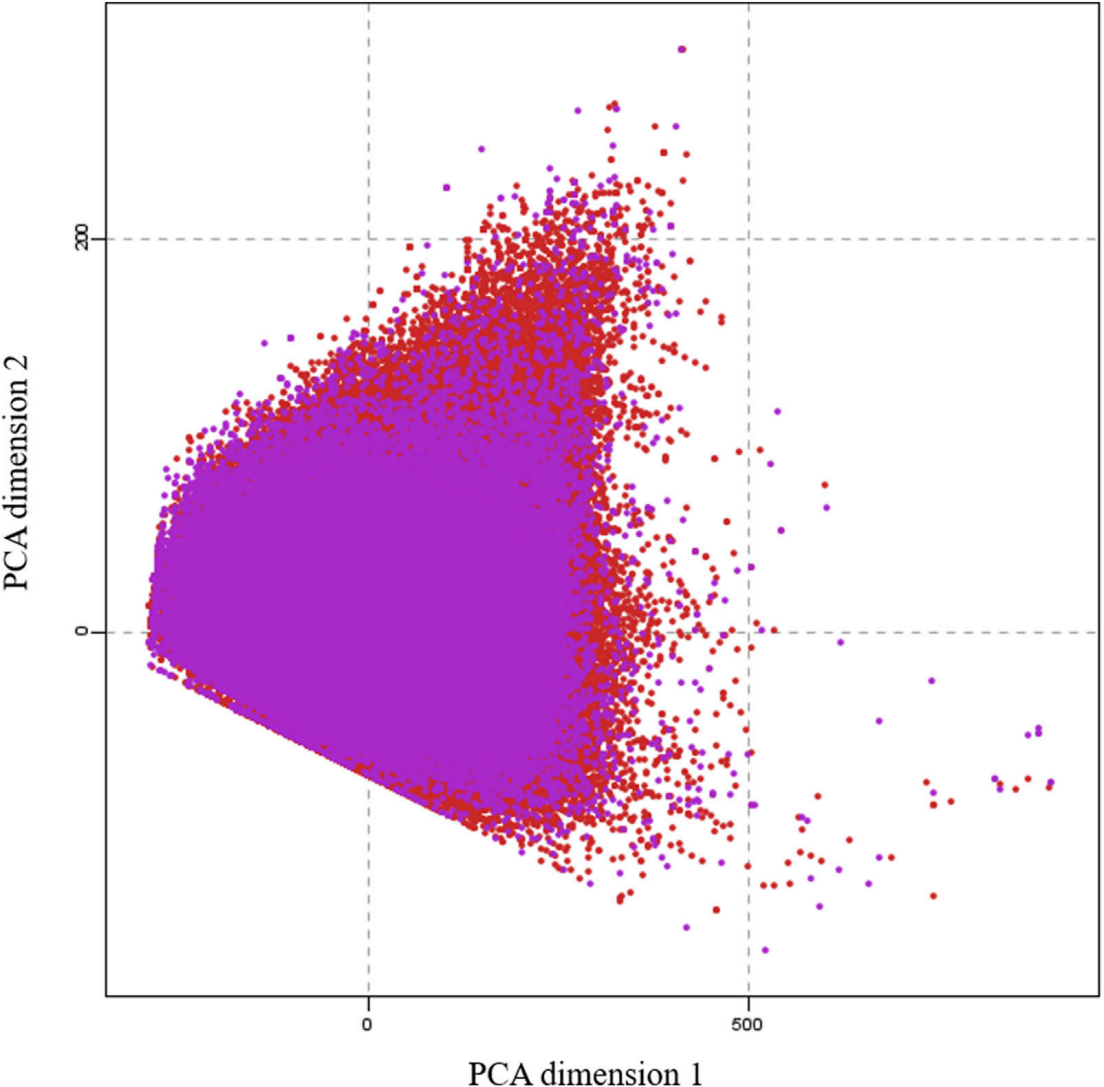
A principal component analysis (PCA) plot, showing the comparison of the chemical space defined by our dataset (purple) and the chemical space represented by CHEMBL25 databases (red).

To generate a training data set for machine learning, we collected 100,000 small molecules to represent the entire druggable chemical space of small molecules from the CHEMBEL-2.5 database (Davies, et al., 2015) after careful removal of salts, ions and small fragments. We considered to perform quantum mechanical calculations for all 100,000 molecules and the collected data would be used for machine learning training. However, it needs 2 years of time to complete all these calculations with our existing computational resources. Thus, the calculations were subjected to three batches. The 31,770 molecules used in this work represent the first batch. To select molecules of this batch, we divided the 100,000 molecules into 10 parts based on their index. Then we selected the third part of each 10,000 molecules by random choice function on the index. To check the trainability of this batch, we have verified by principal component analysis (PCA) that the projection of this batch covers the chemical space of 100,000 molecules. The calculations for the other two batches are in progress. Figure 1 clearly shows that the selected 31770 molecules covered the entire chemical space of 100,000 molecules. This shows that the selected molecules can cover the whole chemical space.

### 2.3 Density functional theory calculations

The 2D structures in Simulation Description Format (SDF) were converted to 3D format using OpenBabel (O’Boyle, et al., 2011) software, and hydrogen atoms were added to all molecules. The 3D geometries of the collected small molecules were optimized using DFT at the B3LYP/6-31G** level of theory with the Gaussian16 package (Frisch, et al., 2016). The optimized geometries were subjected to frequency calculations to confirm that structures were stable on a potential energy surface (PES) at the same level of theory. The frequencies showed that there were no imaginary values, implying that the geometries corresponded to stationary points on the PES. Atomic charges are not observable in experiments or in quantum chemical calculations. Several methods have been suggested to estimate atomic charges. Here, we calculated electrostatic potential (ESP) charges for all atoms in a molecule using the Merz-Kollman method (Chandra Singh and Kollman, 1984) at the B3LYP/6-31G** level. The DFT functional is good in accuracy and predicting ground state properties of molecules compared AM1 method. DFT methods are computationally expensive than AM1 whereas cheaper than MP2 method to perform calculations on large number of molecules.

### 2.4 Machine learning and deep learning

The local environment of an atom in a molecule was described using atomic features. Bonding and neighbor atom information for the atoms in a molecule were extracted with the help of molecular graphs implemented in the MolMod package (Verstraelen, 2019). From the optimized geometries of ESP charges for atoms, bond lengths, bond angles and dihedral angles values were extracted for each molecule in the data set. The local environment around an atom in a molecule strongly influences its atomic charge. Therefore, to train the atomic charge for an atom in a molecule, the atomic features such as atomic number, electronegativity, atomic size, valence, hybridization, aromatic nature, chiral, axial, hydrogen donor or acceptor are first extracted for each atom in a molecule.

**FIGURE 2:**
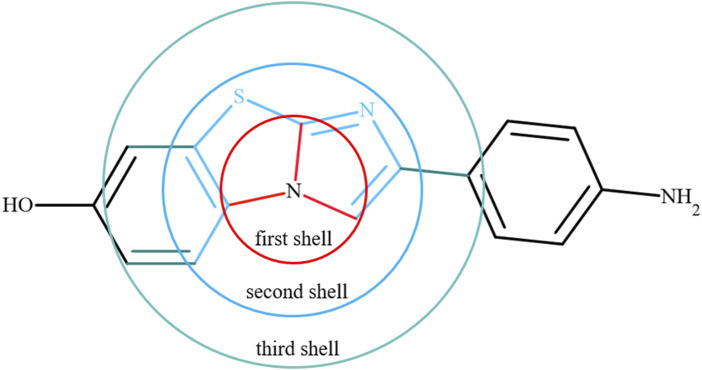
An example to show the chemical environment around a reference atom using the first, second and third shells. Red indicates the first shell, sky blue is the second shell, and Aztec blue represents the third shell around the reference atom.

Next, we included the features of bonding (first shell around the reference atom) information for each atom in a molecule. The local bonded atom information, such as neighboring atoms, number of bonds, bond orders and bond lengths for each atom in a molecule, was extracted from the optimized geometries. The properties of the atoms in the first shell were included using features such as aromaticity, hybridization and the presence or absence of rings, fused rings, and double bonds obtained from structures. We also added information about the atoms present in the second and third shells around the reference atom in a molecule. Overall, the chemical environment was described around one atom in the molecule using the properties of the reference atom and atoms in the first, second and third shells. A schematic of the chemical environment around a reference atom is shown in Figure 2. The information was collected for 1.53 million atoms from 31770 molecules. Accessing such information was not straightforward, and it is not readily available in packages at present. For this purpose, in-house scripts were used to extract all this information.

We applied classification and regression algorithms to train the derived data for small molecules selected from CHEMBEL-2.5. A neural network classifier model was used for training to classify the data. Random forest and neural network regressors were employed to predict numbers for unknown data. We used the Python-based scikit-learn package to construct, train and validate the classification and regressor models (Pedregosa, et al., 2011).

#### 2.4.1 Deep learning

**FIGURE 3:**
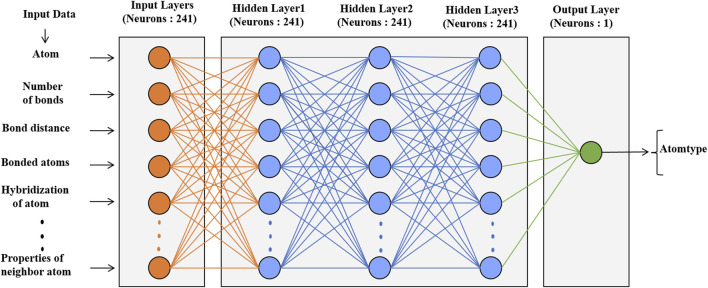
Architecture of the neural network model with the numbers of neurons and atomic descriptors for the prediction of atom types.

The neural network classification model in the scikit-learn package was used for atom types, phase angles and periodicity classification. The architecture of these models is shown in Figure 3; Supplementary Figure S1. The data set had 31,770 molecules resulting in 1.53 million atoms and 4.8 million torsional terms for training atom types and phase angles and periodicities, respectively. The models were trained with a learning rate of 0.001, which controlled the step size in updating the weights, and a default batch size. The default log-loss was used as a loss function. Relu was used as the activation function for the hidden layers, and Adam (Diederik and Jimmy, 2015) a stochastic gradient-based optimizer, was used to update the weights. Similar parameters were used in the prediction of partial charges with the neural network regressor except for the loss function. Mean square error (MSE) was used as the loss function and to validate the model.

#### 2.4.2 Machine learning

**FIGURE 4:**
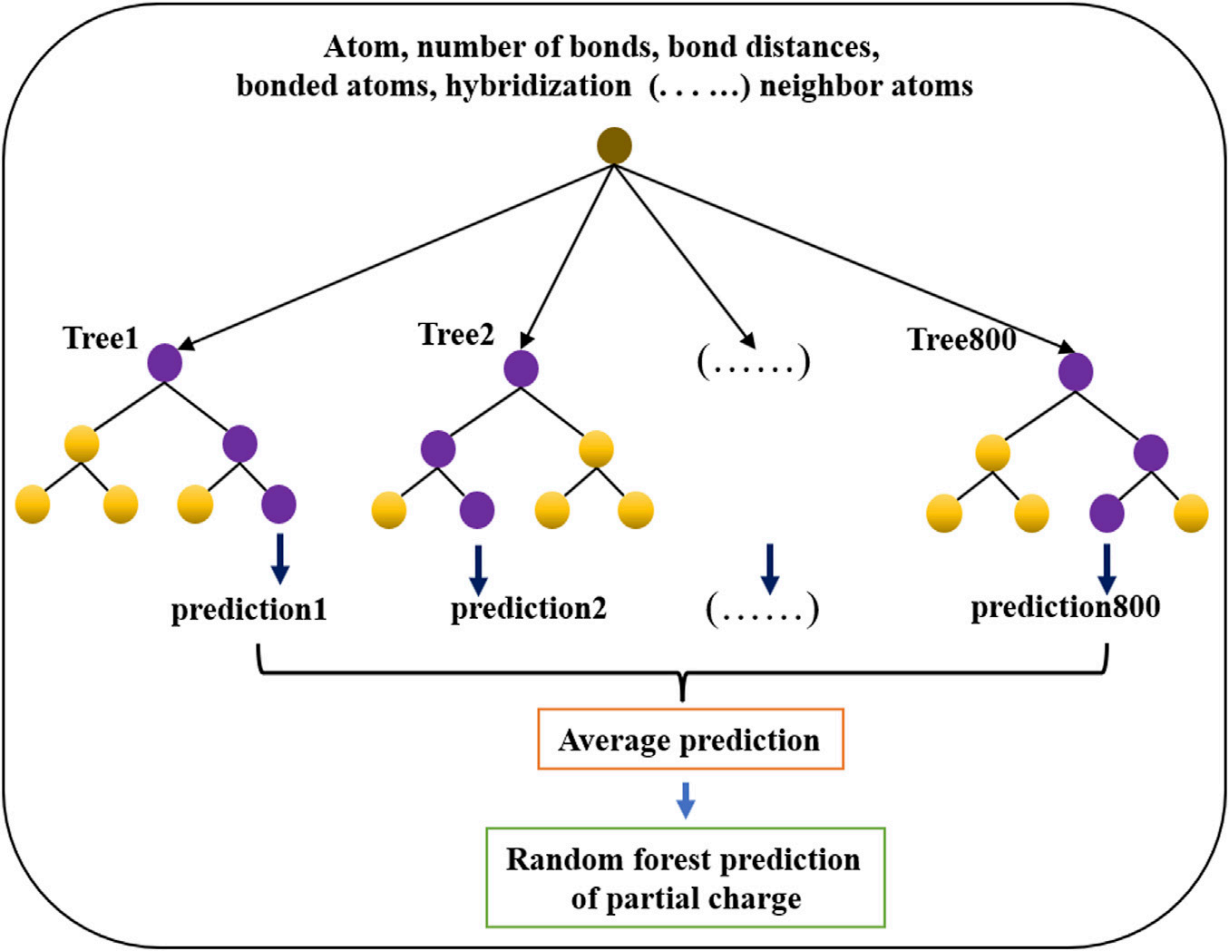
Architecture of the random forest regression model used to predict partial charges for atoms in drug-like small molecules.

The random forest regressor estimator fits trees on various subsamples of a data set and uses averaging to improve the prediction. The random forest regressor model (shown in Figure 4) was used to train and predict the partial charges of atoms in molecules. The model was constructed with 800 trees, and the maximum depth was 100 for each tree. Mean square error was used to validate the regression model. All other parameters were used as default values in the scikit package. In all models, 80% of the data were used for training, and the remaining 20% were used for validation. The parameters of the random forest regressor model were determined by employing k-fold cross validation with k = 5. The mean square error (MSE) was calculated for the predictions in each fold and then averaged.

### 2.5 Molecular dynamics simulations

Free energy calculation methods are generally implemented using the so-called lamination strategy or multistage sampling along a suitably defined chemical coordinate, λ, whereby the system is simulated in an appropriate number, n, of intermediate states corresponding to values of λ between 0 and 1. In this study, small molecules (33 compounds) were selected for solvation free energy calculations. Small molecules were solvated in a cubic box using the TIP3P water model (Jorgensen, et al., 1983). These systems were subjected to energy minimization using the steepest descent method and subsequently equilibrated for 1 ns at 298 K and 1 bar pressure. Velocity rescaling and Parrinello−Rahman algorithms were used to control temperature and pressure in the NPT ensemble (Parrinello and Rahman, 1981; Nose and Klein, 1983; Bussi, et al., 2007). Furthermore, equilibrated solvated structures were simulated for a production run of 1 ns in the NPT ensemble using a 2 fs time step (Berendsen, et al., 1995; Lindahl, et al., 2001; Hess, et al., 2008). The particle mesh Ewald method was used to calculate the electrostatic interactions with an interpolation order of 4 and a grid spacing of 1.6 Å (Essmann, et al., 1995). Bonds between hydrogen and heavy atoms were constrained at equilibrium bond lengths using the LINCS algorithm (Hess, et al., 1997). All simulations were performed using the GROMACS-2020 package.

**FIGURE 5:**
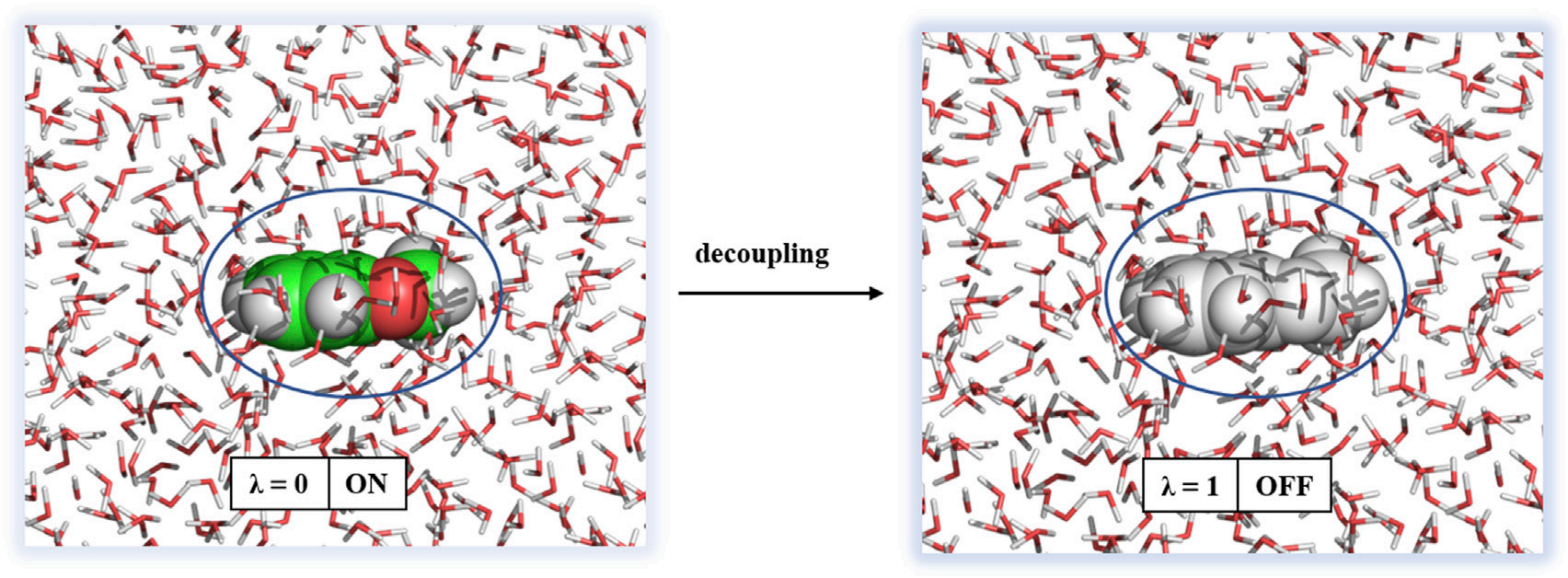
Decoupling of a ligand from solvation. Water molecules are shown in sticks and ligand as spheres.

All solvation free energy calculations were performed by decoupling the ligand from the solvent environment. The initial conformation of the ligand in solvent was taken from the final snapshot of the 1 ns simulation. Decoupling of the ligand from solution was performed by turning off Coulombic interactions and subsequently van der Waals interactions. The approach of solvation free energy calculation is shown in Figure 5.

The Coulombic interactions were turned off by changing λ from 0 to 1 with a step size of ∆λ = 0.25, and the van der Waals interactions were unperturbed. Then, the van der Waals interactions were turned off with nonuniformly distributed values of λ (0.05, 0.1, 0.2, 0.3, 0.4, 0.5, 0.6, 0.65, 0.7, 0.75, 0.8, 0.85, 0.9, 0.95, and 1.0). Therefore, a total of 20 windows, each 1 ns, were employed for decoupling of the ligand from solution. The free energy difference between two end states was calculated using the Bennett Acceptance Ratio (BAR) method (Bennett, 1976) and the following equation:

$$\langle \frac{1}{1+\exp\left\{\beta \left({\Delta U}_{ij}ᅳ\;\Delta G\right)\right\}}\rangle i=\langle \frac{1}{1+\exp\left\{\beta \left(ᅳ\;{\Delta U}_{ij}+\Delta G\right)\right\}}\rangle j$$ (2)

where β is the reciprocal of the thermodynamic temperature, ΔG is the free energy difference between states i and j, and ΔU_*ij*_ = U_*j*_ - U_*i*_ is the potential energy difference.

### 2.6 Protein–ligand simulations

The crystal structure of the protein kinase, covid19 (main protease) and factor-IX with cocrystal ligand were taken from the protein data bank (PDB id: 4XUF, 7L10 and 5TNT). Protein structures were prepared by correcting the bond orders, adding missing hydrogens and optimizing H-bonding with protonation states of residues at pH 7.0 using protein preparation wizard (Sastry et al., 2013). The complex was solvated in a cubic box with a TIP3P water model. The total charge of the proteins was neutralized by inclusion of Na^+^ and Cl^−^ ions. The AMBER99SB-ILDN force field was used for the proteins. The force field parameters for the cocrystal ligands were generated using generalized amber force fields (GAFF) and machine learning force field for the comparison. All solvated the protein and ligand complexes were subjected to energy minimization using steepest decent method. Temperature and pressure controls were imposed using the V-rescale and Parrinello-Rahman algorithms with 298 K and 1 bar, respectively (Parrinello and Rahman, 1981; Nose and Klein, 1983; Bussi, et al., 2007). The simulations were carried out with a time step of 2 fs for 1 ns to equilibrate the systems in the NPT ensemble. The production run was performed for 250 ns for each complex using a time step of 2 fs in NPT ensemble. The interpolation order of 4 and a grid spacing of 1.6 Å were used in the electrostatic calculations using particle mesh Ewald method (Essmann, et al., 1995). LINCS algorithm have used to constrain the bonds of hydrogens with heavy atoms (Hess, et al., 1997).

## 3 Results and discussion

### 3.1 Prediction of partial charges

**FIGURE 6:**
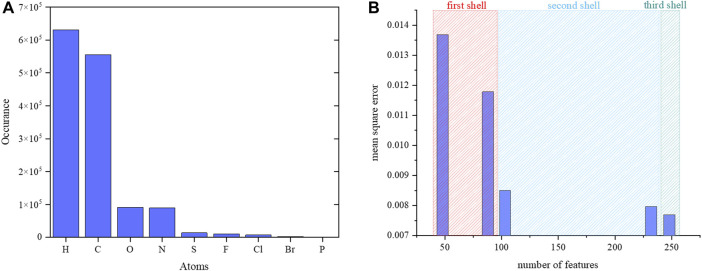
**(A)** Number of samples per element in the data set. Numbers for phosphorous atoms are not visible in the graph as very few phosphorus atoms were present in the data. **(B)** The calculated MSE vs. number of atomic features for a reference atom.

The number of samples per element presented in the data set is shown in Figure 6. Each atom has its local chemical environments and reference partial charge in the data. The calculated MSE in Supplementary Table S1 shows that the random forest regressor is slightly better than the neural network regressor. Therefore, a random forest regression model was adopted for further validation. The MSE was optimized by increasing the number of descriptors for each atom in the data set. The descriptors were atoms and their properties in the first, second and third shells around a reference atom. The MSE with respect to the number of descriptors is shown in Figure 6. The addition of the chemical environment reduced the MSE value for the random forest regression model. The performance of the random forest regression model was best when all atoms and their properties were included in the three shells. In previous study also, it was shown that random forest regression produces reliable results compared to other machine learning algorithms. Previous study randomly collected the data for 10000 and 7,000 molecules from ATB (automated topology builder) and PRODRG servers. ATB applies symmetry-based averaging of atomic charges based on the ESP charges from B3LYP/6-31G* calculations for small molecules with the number of atoms less than 40 otherwise it carried out semiempirical calculations to generate the charges. Whereas in this study, we have performed calculations at B3LYP/6-31G* for all the molecules which are having more than 40 number atoms in addition smaller size molecules. The considered data for the training of random forest regression model has the molecules with atoms range from 10 to 120. The number of data points and features are used in the training of our charge model (241 features) is higher than the previous study (61 features). The features include the bond orders, bond lengths, hybridizations and electronegativities for neighbor atoms to provide the chemical environment around an atom whereas the previous study does not consider them.

**FIGURE 7:**
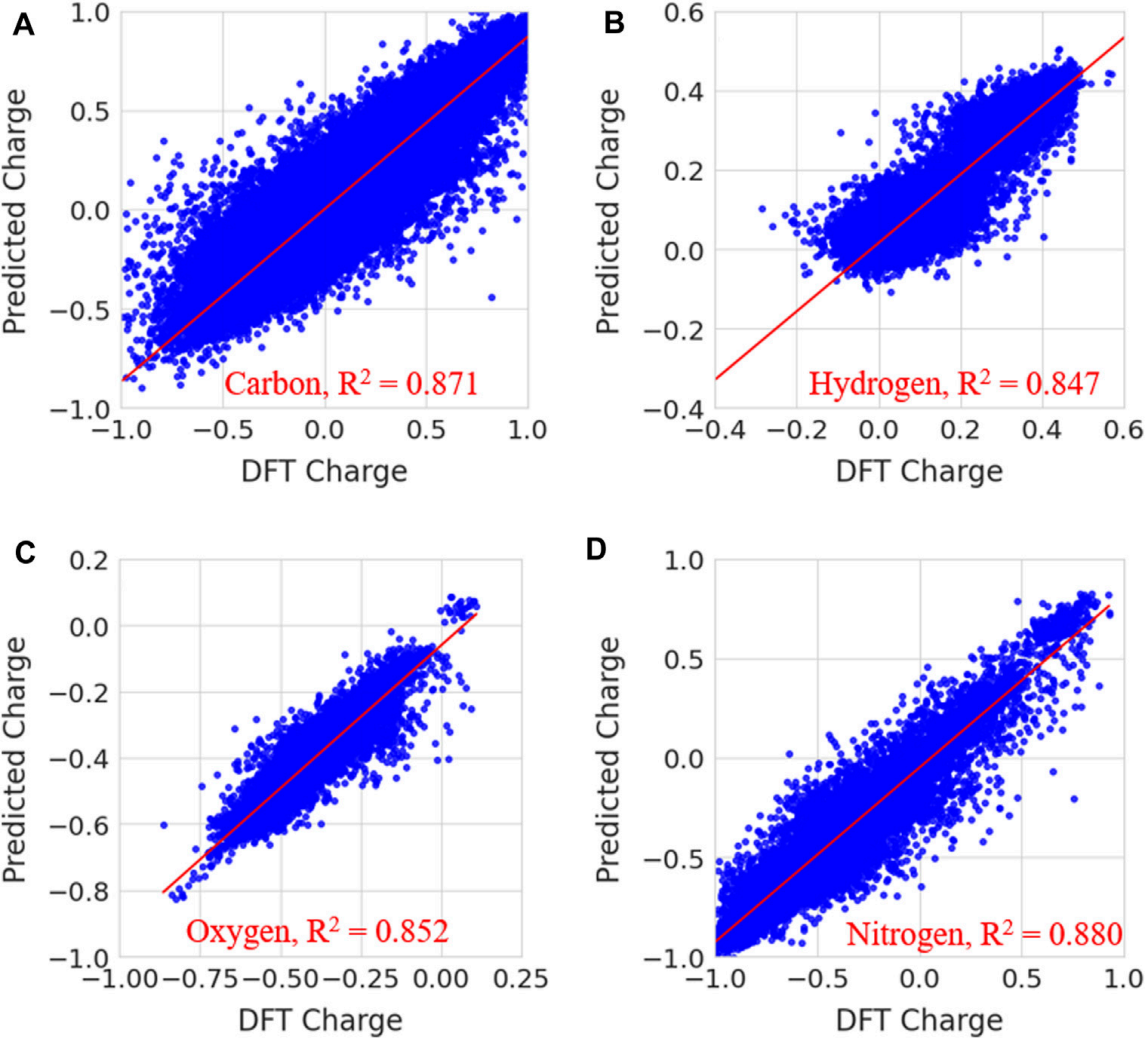
Prediction of partial charges for **(A)** carbon **(B)** hydrogen, **(C)** oxygen and **(D)** nitrogen atoms in the test data set using a random forest regression model.

In Figure 7, the predicted charges were fitted to reference charges for elements including carbon, hydrogen, nitrogen and oxygen. The same plots for other elements, such as sulfur, fluorine, chlorine, bromine and phosphorous, are given in Supplementary Figure S2 of the Supplementary Information. Notably, the majority of the predicted charges were similar to the reference values. The calculated coefficient of determination (R^2^) and MSE values for carbon, hydrogen, oxygen, nitrogen, sulfur, fluorine, chlorine, bromine and phosphorous are presented in Supplementary Table S2. The calculated coefficient of determination (R^2^) values for carbon, hydrogen, oxygen, nitrogen, sulphur, fluorine, chlorine, bromine and phosphorous are 0.871, 0.847, 0.852, 0.880, 0.977, 0.632, 0.805, 0.714 and 0.664, respectively. MSE values are 0.0148, 0.001, 0.002, 0.013, 0.004, 0.0003, 0.004, 0.001 and 0.027 for carbon, hydrogen, oxygen, nitrogen, sulphur, fluorine, chlorine, bromine and phosphorous, respectively. The prediction accuracy was less for fluorine, bromine and phosphorous than for other elements. This may have been due to a smaller number of samples in the data. The prediction accuracy for atoms such as C, H, O, N, S, P, F, Cl and Br is low when compared to previous study. Because the number of date points and data for each atom is different and it increases variance in the atomic charges thus makes difficulty in the prediction. It is difficult to compare the charges from our charge model with other methods because the atomic charges for a molecule using QM calculations are often sensitive to functional and fitting method which are used to generate ESP charges. The charges from different fitting methods are not same for a molecule. However, we have provided the comparison of our charge predictions with ESP charges of ATB, QM and AM1-BCC methods for one molecule in Supplementary Table S3. It can be clearly noted that charges in all these methods are not same. The quantity of atomic charges is different in each method whereas the sign (+ or -) is same in the case of all atoms. In order to understand the atomic charges produced from random regression model, the calculated solvation free energies for molecules using different charge methods are compared with experimental values in the validation section.

**FIGURE. 8:**
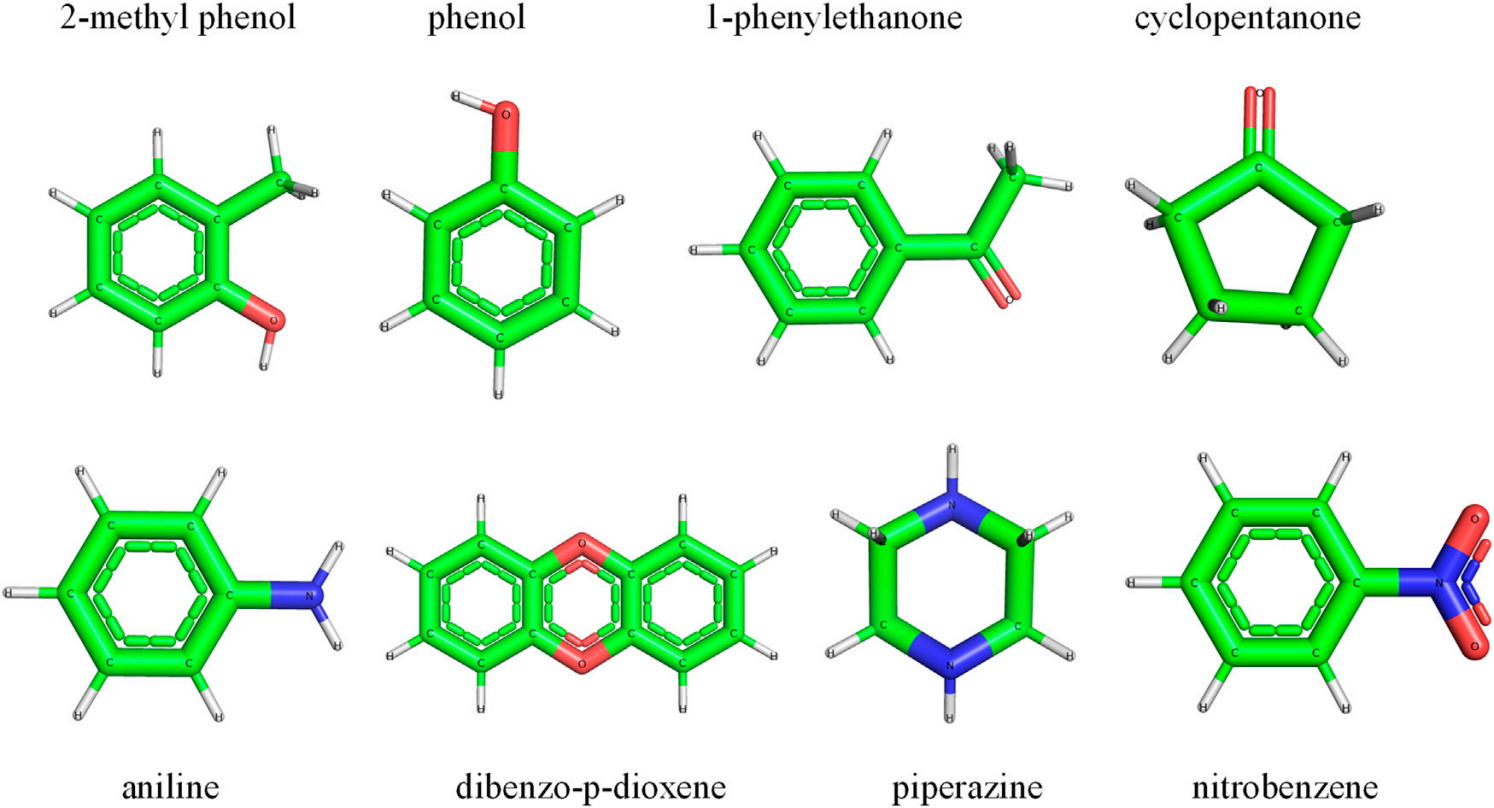
A few small molecules were selected from test set-2 for the validation of the random forest regression model.

**FIGURE 9:**
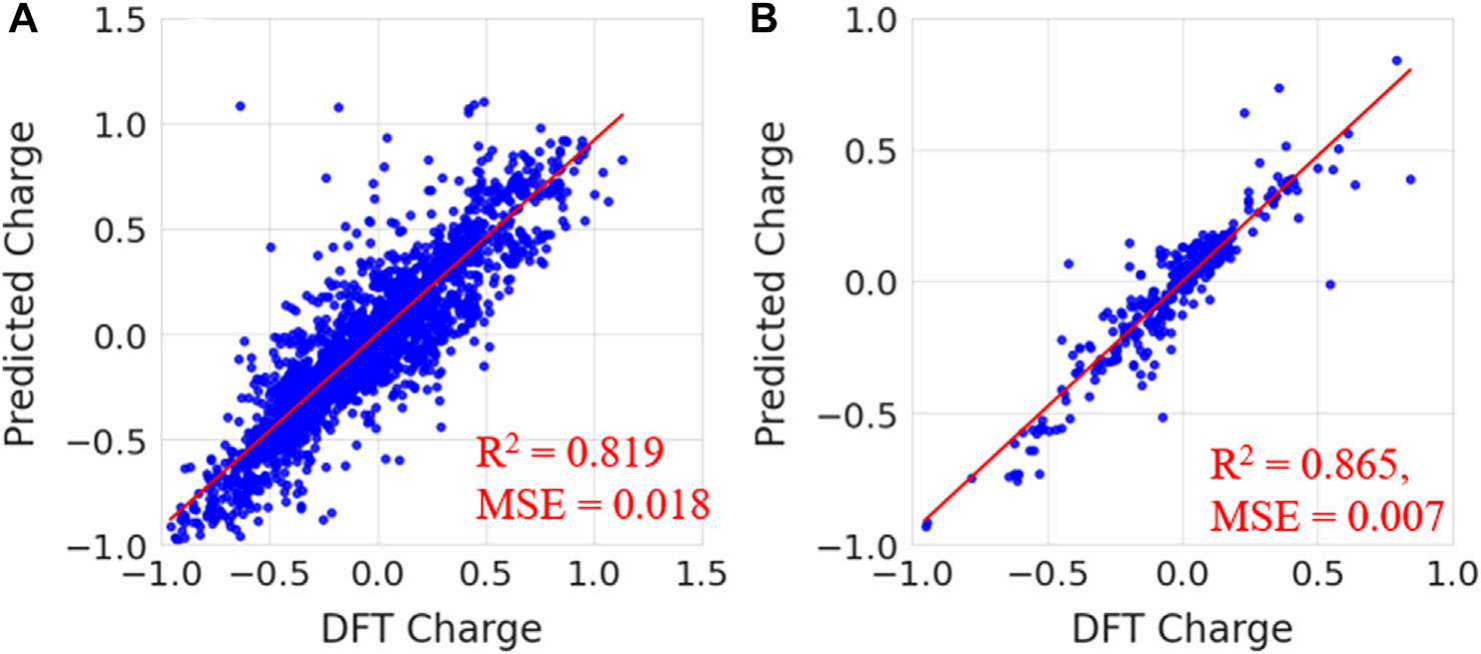
Predicted charges vs. DFT charges for **(A)** test set-1 from the drug-induced liver injury database and **(B)** test set-2 for known small molecules.

To validate, the performance of the trained random forest regression model was tested on two external test sets. Test set-1 consisted of 100 molecules that were randomly selected from the drug-induced liver injury database. This database consists of FDA approved drugs that are shown to be toxic to the liver. Test set-2 considered 33 molecules that had experimental solvation free energies in the literature. We have tested the charge model on two different datasets (i) first dataset is having molecules which contains atom numbers range from 20 to 87 (ii) second dataset contains molecules with atom number range from 9 to 24. The small molecules in test set-2 consisted of various electron-donating and electron-withdrawing functional groups. Eight small molecules from test set-2 are shown in Figure 8. The predicted charges are plotted against DFT charges for both test sets and displayed in Figure 9. The R^2^ and MSE values reveal that the prediction accuracy for the test sets was high. The predicted values for a few molecules are compared with ESP charges obtained from DFT calculations in Table 1 and Supplementary Table S4. Table 1 shows that the predicted values were close to the DFT charges. The random forest regressor gave the correct sign (+ or -) and values similar to the ESP charges. It was evident that the random forest regressor model produced can work for small molecules as well as for large size molecules.

**TABLE 1:** Comparison of predicted partial charges from the random forest regression model with DFT charges.

Cyclopentanone			Aniline		
Atom	Random forest	DFT	Atom	Random	DFT
O	-0.56910	-0.49271	N	-0.84978	-0.78174
C	-0.06156	-0.04073	C	0.18780	0.35203
C	-0.05696	-0.04086	C	-0.29226	-0.25455
C	-0.15291	-0.19790	C	-0.25365	-0.25456
C	-0.23938	-0.19782	C	-0.06735	-0.10162
C	0.59172	0.54435	C	-0.08070	-0.10157
H	0.02416	0.03785	C	-0.08311	-0.15603
H	0.04566	0.02639	H	0.16222	0.14008
H	0.04022	0.03786	H	0.14058	0.14009
H	0.05218	0.02643	H	0.13362	0.11626
H	0.08876	0.06753	H	0.13362	0.11625
H	0.08969	0.08105	H	0.13362	0.11343
H	0.07499	0.06753	H	0.36227	0.33594
H	0.07256	0.08101	H	0.37313	0.33595

However, it is noted that machine learning charge model can assigns wrong sign (+ or -) for aliphatic carbon atoms compared to DFT ESP charges. For example, we considered the charges for aliphatic molecule (1-Octanol) which are generated using random forest model and DFT calculation Supplementary Table S5. It can be seen that C3, C4, C5, C6 and C7 have positive atomic charge in random forest prediction. Whereas the atoms C3, C5 and C6 are negative and C4 and C7 are having positive partial charge in the case of ESP from DFT calculations. Because all C3, C4, C5, C6 and C7 are sharing similar kind of bonding environment thus random forest predicts positive charges for them. The prediction of charges can be improved by adding a greater number of diverse aliphatic molecules into the data set to reproduce the ESP of DFT.

Existing small molecule force field generate programs such as antechamber, CGenFF, ATB and PRODRG produces charges based on ESP. Antechamber program uses ESP charges from quantum calculation and produces restrained electrostatic potential (RESP) using least square fitting method. We have used antechamber to generate RESP charges for small dataset of 2,700 molecules to train using random forest regression model. The obtained charge model shows the MSE of 0.027 on the test set. We have predicted the RESP charges for testset-1 and testset-2 datasets. The calculated coefficient of determinations are 0.71 and 0.61 Supplementary Figure S3. The model shows promising result though the trained on a smaller number of atoms. The model can be improved a lot by incorporating a greater number of atoms into training set. We will develop a RESP charge prediction model using greater number of molecules in the near future.

### 3.2 Atom type prediction

Categorizing the atoms in a molecule into atom types is useful to assign the force field parameters. Antechamber programs were used to generate atom type data for atoms in the molecules. The trained neural network classifier model performed well with 98% accuracy on the test data set. The model identified the atom types based on the provided local chemical environment around a reference atom. The calculated confusion matrix produced precision, recall, F1-score and accuracy for each atom-type prediction. The model could identify only the following atom types: C, H, O, N, S, P, F, Cl, and Br. The prediction accuracy for each atom type is given in Table 2. In Table 2, from c to cy, from h1 to hx, from n to nq, from o to os, from p3 to py, from s to sy, f, cl and br are atom types for C, H, N, O, P, S, F, Cl, and Br, respectively. The definition for each atom type is similar to the generalized amber force field (GAFF). The model clearly identifies all types of H, O, F, Cl, and Br atoms with 100% accuracy. Additionally, the sulfur and phosphorus atom type prediction accuracy was 100%. The most common aliphatic, cyclic and aromatic atom types of carbon (c, c1, c2, c3 and ca) and nitrogen (n, n1, n2, n3, na and nb) were predicted with accuracy above 95%. The predictions were the least accurate for atom types cf and nf. However, the model assigns incorrect atom type in the case of sp^2^ carbons such as cc, cd, ce, cp and cf only with another sp^2^ carbon type (cc, cd, ce, cp and cf). All sp^2^ carbons (nitrogen’s) have the same van der Waals parameters in GAFF. The same is true in the case of sp^3^ carbons and nitrogens. In our force field, atom type prediction is used to assign van der Waals parameters for the atoms in a molecule. Therefore, even the incorrect prediction of atom types would not effect the force field parameters.

**TABLE 2:** Accuracy of the prediction of atom types using a neural network model.

Atomtype	Precision	Recall	f1-score	Atomtype	Precision	Recall	f1-score
br	1	1	1	n	1	0.99	0.99
c	1	1	1	n1	0.99	0.99	0.99
c1	0.99	0.97	0.98	n2	0.99	0.96	0.97
c2	0.96	0.98	0.97	n3	1	0.98	0.99
c3	1	1	1	n4	1	0.67	0.8
ca	0.96	0.99	0.98	na	0.99	0.98	0.98
cc	0.82	0.66	0.73	nb	0.92	0.97	0.95
cd	0.72	0.67	0.69	nc	0.75	0.44	0.56
ce	0.72	0.8	0.76	nd	0.74	0.87	0.8
cf	0.58	0.45	0.51	ne	0.66	0.8	0.72
cg	0.7	0.93	0.8	nf	0.17	0.05	0.08
ch	0.43	0.1	0.16	nh	0.96	0.99	0.98
cl	1	1	1	nj	1	1	1
cp	0.57	0.79	0.66	nm	1	1	1
cq	0	0	0	no	1	1	1
cv	0.5	0.5	0.5	np	1	1	1
cx	1	1	1	nq	1	1	1
cy	1	1	1	o	1	1	1
f	1	1	1	oh	1	1	1
h1	1	1	1	op	1	0.83	0.91
h2	1	0.93	0.96	os	1	1	1
h3	1	1	1	p5	1	1	1
h4	1	1	1	py	1	1	1
h5	1	1	1	s	1	1	1
ha	1	1	1	s4	1	1	1
hc	1	1	1	s6	1	1	1
hn	1	1	1	sh	1	1	1
ho	1	1	1	ss	1	1	1
hs	0.97	1	0.98	sx	1	1	1
hx	1	0.25	0.4	sy	1	1	1

To assess the accuracy, the model was used to predict the atom types for a few small molecules, as shown in Figure 8. The predicted atom types were compared with antechamber-produced atom types; the results are presented in Table 3 and Supplementary Table S6. Table 3 and Supplementary Table S6 show that the atom types predicted by the neural network model were in good agreement with the predictions of the antechamber program. The neural network classifier accurately identified the atom types and their chemical environments. This ensured that the model successfully assigned atom types for small drug-like molecules.

**TABLE 3:** Comparison of the atom types predicted by the neural network model and antechamber program.

Cyclopentanone			Aniline		
Atom	NN model atom type	Antechamber atom type	Atom	NN model atom type	Antechamber atom type
O	o	o	N	nh	nh
C	c3	c3	C	ca	ca
C	c3	c3	C	ca	ca
C	c3	c3	C	ca	ca
C	c3	c3	C	ca	ca
C	c	c	C	ca	ca
H	hc	hc	C	ca	ca
H	hc	hc	H	ha	ha
H	hc	hc	H	ha	ha
H	hc	hc	H	ha	ha
H	hc	hc	H	ha	ha
H	hc	hc	H	ha	ha
H	hc	hc	H	hn	hn
H	hc	hc	H	hn	hn

### 3.3 Prediction of phase shift angles for dihedral terms

The phase shift angle is involved in the dihedral energy term, and it is important to calculate the energy contribution from the dihedral energy term to the total potential energy. Each dihedral term had a specific phase angle value and was restricted to the range between 0° and 180°. The 4.8 million dihedral terms in 31770 molecules were extracted along with their phase angles. Atomic descriptors were generated for the atoms involved in each dihedral angle. The calculated dihedral angle values were also included to train the neural network classifier to predict phase angles of 0° and 180°. The trained model classified the test data set as 0° and 180° with 94% accuracy. The predicted values were well correlated with the parameters generated by the antechamber program. The incorrect prediction of phase shift angle for dihedral angle can produces the unwanted angle rotations or restrictions thus causes changes in the conformation of ligand compared to GAFF. The phase shift angle is important to retain the planarity of aromatic ring and conjugated groups in the molecules. Our phase angle model predicts accurately for these kinds of molecules and retained the planarity of molecules. However, phase shift angle model (accuracy 94%) has to be improved further to avoid the unfavorable conformational changes in the molecules by increasing the number of data points and feature incorporation in the training dataset.

Neural network model training was conducted with the same atom features for the inclusion of phase angles for the prediction of periodicity for dihedral terms. The model performed the prediction with 93% accuracy. The accuracy for classification of terms with periodicity 2 and 3 was 96% and 95%, respectively. The predicted periodicities were retained the structures of aromatic and other types of molecules. The predicted phase angles and periodicities were compared with the antechamber-generated values; they are shown in Supplementary Table S7.

### 3.4 Generation of topology for a molecule

**FIGURE 10:**
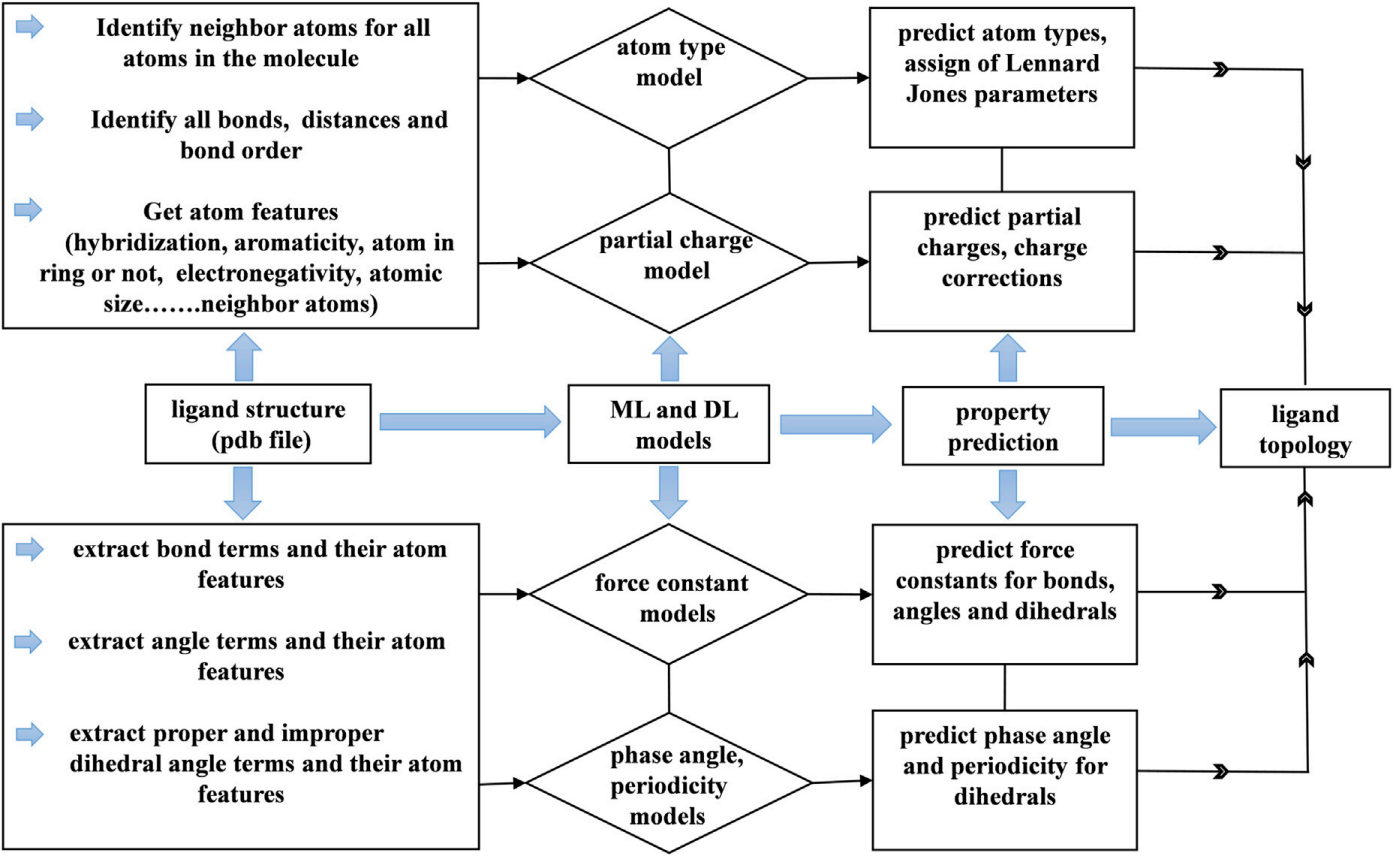
Workflow of the generation of the topology for small drug-like molecules using machine learning and deep learning models.

The concept of using AI algorithms was to generate parameters and topology for small molecules that generally did not have parameters in conventional force fields. Few commercial and noncommercial software packages, such as ATB (Stroet, et al., 2018), antechamber, CGenFF and PRODG (Schüttelkopf and Van Aalten, 2004), are available to generate force field parameters for small molecules. We generated topologies for small drug-like molecules using machine learning models to predict atom types, DFT-based partial charges, phase angles, periodicity and force constants for bonds, angles and dihedrals. The work flow is shown in Figure 10. In this study, flow, data collected from a molecule were used to perform predictions by employing machine learning and deep learning models. The collected information and predicted data were used to generate topologies in the format of used in most MD simulation programs, such as GROMACS and NAMD.

Topology generation started with the prediction of atom types for a given molecule. The small molecule force fields like GAFF have limited number of atom types. Each atom type has corresponding Lennard jones parameters. These parameters assignment depends on the atom type. The atom type prediction was done by our model with 98% accuracy with respect to GAFF. Based on atom types, Lennard jones parameters were assigned accordingly. Lennard jones parameters were taken from the GAFF force field. This gives the correct assignment of Lennard jones parameters to the atoms in a molecule. Next, the partial charge model predicted atomic charges for all the atoms in a molecule. The sum of the predicted atomic charges was not equal to the formal charge of the molecule. Therefore, charge correction was applied in such a way that the sum of the predicted charges was subtracted from the formal charge of the molecule, and the difference was distributed among all the atoms to make the total charge of the molecule equal to the sum of the predicted atomic charges. Furthermore, a list of the bonded atoms and bond lengths was calculated, and the bond force constants were predicted with the aid of a trained model. Here, bond lengths from the structure were used as equilibrium distances for bonds. Subsequently, the angles and dihedral terms were added to the topology in the respective sections. Then, the nonbonded 1, 4 pairs for the molecule were generated by taking the first and fourth atoms in dihedral angle terms.

Next, we generated improper dihedral angle terms for the topology file. No tool was used to identify the improper dihedral angles in small molecules other than current force field generation programs. In general, improper angles are intended to maintain the planarity of aromatic and conjugated molecules. Aromatic and conjugated molecules are predominantly involved with carbon atoms. Three atoms are bonded to carbon atom that is involved in a double bond. We generated a list of improper dihedral angles based on the number of atoms bonded to carbon atoms and with the extraction of their neighboring atoms. Eventually, we generated force field parameters for drug-like molecules within a minute of CPU time. The correct assignment of partial charges and van der Waals parameters to the atoms enables the molecules to interact with environment such as water and protein through nonbonded interactions. The atomic features are the important in order to understand the chemical environment which effects partial charges, atom type and phase angle predictions. The user has to provide proper molecule structure by adding all hydrogens to heavy atoms otherwise user may end up with assigning of incorrect parameters which can collapses molecule structure.

### 3.5 Validation of the force field

#### 3.5.1 Solvation free energies

**FIGURE 11:**
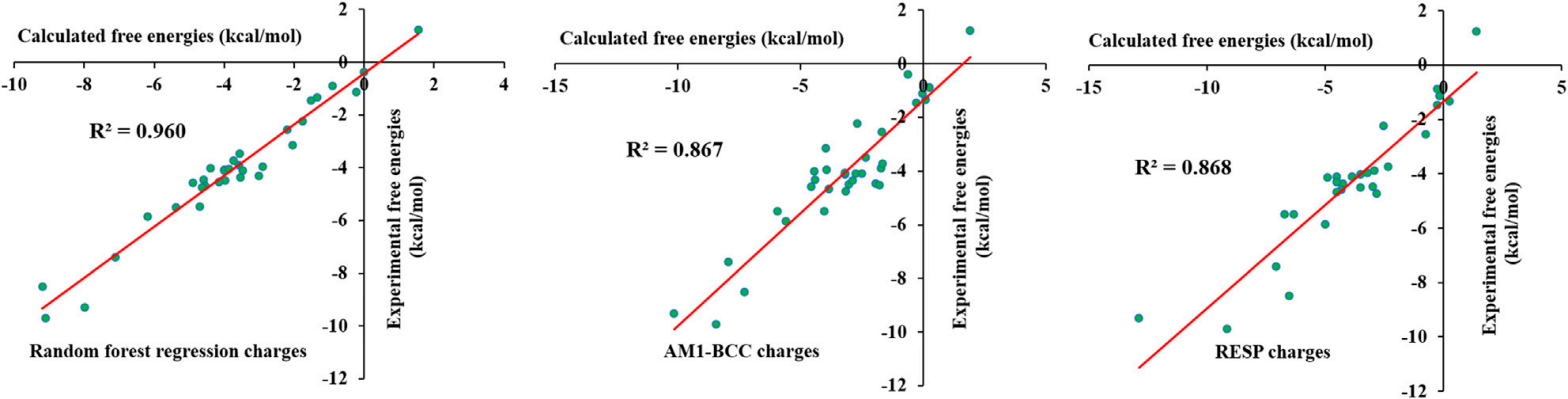
Comparison of experimental solvation free energies of small molecules in test set-1 with random forest regression, AM1-BCC and RESP charges.

To verify the predicted partial charges and other force field parameters, solvation free energies were calculated for 33 selected small molecules using the λ-coupling method. This method is reliable and accurate in the calculation of solvation free energies and has been used to calculate protein–ligand absolute binding free energies. The selected 33 molecules contained various functional groups, including alcohol, thiol, amide, amine, aldehyde, ketone, nitro, nitrile, and methyl groups and halogens. Aliphatic chains, aromatic rings and cyclic rings were also present in the chosen molecules. The calculated free energies were compared with the experimental free energies; the results are shown in Supplementary Table S8. The calculated values were in close agreement with the experimental free energies. The calculated values were within 2 kcal/mol error from experimental free energies except for several molecules. To obtain reasonable free energy values, we introduced charge corrections to the atoms involved in specific bonds. This was done based on previous studies where localized bond charge corrections were added to improve the solvation free energies of small molecules (Dodda, et al., 2017). Localized bond charge corrections for few bonds were taken from the literature (Dodda, et al., 2017), and others were calculated based on a trial and error approach. The charge corrections for specified bonds are shown in Supplementary Table S9. Charge corrections were performed for aliphatic, cyclic and aromatic bonds. The introduction of charge corrections significantly improved the free energy values, which were similar to the experimental numbers. The calculated values are shown in Supplementary Table S8, and they reveal that the calculated values were similar to the experimental values. It can be seen that though the incorrect assignment of atomic charges for carbon atoms in 1-Octanol produces solvation free energy close to experimental value. Figure 11 shows that the R^2^ value reached 0.960. Thus, the corrected charges accurately described the interaction of molecules with the water environment. We have also compared the calculated solvation free energies from AI force field, AM1-BCC/GAFF and RESP/GAFF with experimental values. The calculated solvation free energies for AM1-BCC/GAFF and RESP/GAFF were taken from the literature (Shivakumar, et al., 2009) and given in Supplementary Table S8. The calculated coefficient of determination for AI force field, AM1-BCC/GAFF and RESP/GAFF are 0.960, 0.867 and 0.868, respectively. The results shows that AI force field outperforms the other methods in reproducing the experimental values. However, further AI force field has to be tested on large number of molecules and compare with experimental values. Overall, the machine learning force field successfully reproduced the experimental free energies, revealing that the force field was accurate and reliable.

#### 3.5.2 Protein–ligand interactions

**FIGURE 12:**
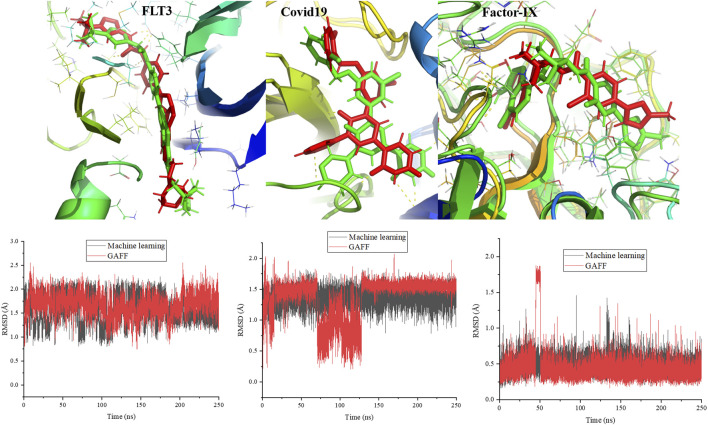
Simulated structures of proteins related to kinase, covid19 and factor-IX at 250 ns are compared between machine learning force field and GAFF. The calculated RMSD for the ligands in protein-ligand complex throughout the simulation. Ligand represent in green and red color corresponds to machine learning and GAFF, respectively.

To validate the force field parameters generated by the machine learning force field, MD simulations were performed for protein and ligand complexes and then compared with the results of simulations with the GAFF. The complexes were stable throughout the simulations, and the final snapshots at 250 ns are shown in Figure 12. The ligand was composed of aromatic and nonaromatic rings. There were no distortions in the ligand structure, and it was stable in the pocket. The surrounding interacting residues for the ligand were the same in the cases of the machine learning and amber force fields. However, the atoms involved in hydrogen bond formation were different in the final snapshots from both force fields. Additionally, the ligand conformation was slightly different in the case of the machine learning force field compared to the GAFF (Figure 12). The calculated root mean square deviations (RMSDs) for the ligand throughout the simulations are presented in the Figure 12. Notably, structural changes in the ligand were not significant in either force field. The average RMSDs of the ligand with respect to the starting conformation were 1.57 and 1.67 Å for the machine learning and GAFF force fields, respectively. In addition to protein kinase, we have performed simulations of 250 ns for the proteins such as covid 19 (main protease) (pdb id:7L10) and factor-IX (pdb id: 5TNT). In 5TNT, the ligand binds at the surface of protein, however, it is stable at the binding site throughout the simulation. We compared the snapshots of AI force field and GAFF and it is shown in Figure 12. The structure of ligand at the binding is not same in both force field, however the difference is marginal. The ligands are stable at binding site through interactions with the residues of protein. The plots show that the there is no significant structural changes in the ligand with respect to RMSD values.

The stability of complexes derived from the interaction energy was calculated and shown in the supporting information. The interaction energy was clearly less for the machine learning force field than the GAFF. Furthermore, the electrostatic and van der Waals energy contributions to the total interaction energy were calculated, and the results showed that electrostatic interactions were responsible for the difference in the interaction energies. The average electrostatic interaction energies between the protein and ligand were -17.4 and -30.5 kcal/mol for the machine learning and GAFF force fields, respectively. The electrostatic interaction energy was different due to variations in atomic charges between the machine learning force field and GAFF. The difference clearly shows that the machine learning force field should be improved to minimize the differences in the energies and conformations of the ligand compared to those obtained using the GAFF. We expect to study ways to improve the force field by including more data in the training data set to maximize interactions between proteins and ligands and enhance the prediction of phase angles.

## 4 Conclusion

A force field for small drug-like molecules was generated using machine learning and deep learning techniques. The random forest regression based charge model generates quality atomic charges comparable to DFT based ESP charges which are suitable for molecular dynamics simulations. In addition to the charge model, we developed AI-based models to predict atom types, force constants, phase angles and periodicities for dihedral terms. The classifications of atom types, phase angles and periodicity were achieved successfully with accuracies of 98, 94 and 93%, respectively. The AI models could able to predict charges and atom types with high accuracy based on the provided atomic chemical environment through features around a reference atom. Using all these models, we developed a module in the pharmulator™ platform that generated topology files for small molecules in GROMACS and NAMD formats to perform molecular dynamics simulations. The code generates quality atomic charges and other compatible force field parameters within a minute of time. The generated force field parameters for small molecules reproduces the experimental solvation free energies with coefficient of determination value of 0.96. The calculated free energies are better reproduced than AM1-BCC and RESP charges. Further, the calculated structural changes in ligand molecules at protein binding sites are comparable with GAFF results. Overall, the results clearly revealed that the force field generated by machine and deep learning techniques was accurate and reliable for use in molecular dynamics simulations of small molecules as well as for complexes of proteins and ligands. The machine learning charge model differs from AM1-BCC and CGenff methods in terms of methodology and level of theory used to generate atomic charges. This method could optimize the efficiency and accuracy of calculations to produce reasonable ESP charges. Also, DFT calculations to obtain ESP charges were included at additional computational cost, which increased with the size of the molecule. Therefore, the rapid prediction of accurate ESP partial charges, within a minute of time and without quantum mechanical calculations, would be very helpful in the drug discovery process.

However, AI based force field models may have certain limitations that it assigns incorrect sign (+ or -) for aliphatic carbon atoms compared to ESP charges from DFT. In some cases, the prediction phase angle for dihedral angle can be incorrect that may introduce flexibility or rigidity in the molecules. These limitations can be overcome by adding large number of diverse aliphatic molecules into training data set. The increasing of data points and number of features for training of phase angle model would improve the accuracy to overcome the limitations.

Generation of force field parameters for ligand molecules is useful to perform molecular dynamics simulations to analyzes the interactions and to estimate binding free energy in implicit and explicit water environment. The estimation of atomic partial charges of the small molecules to calculate molecular interaction fields (MIFs) is an important process in field-based quantitative structure-activity relationship (QSAR) (Mittal et al., 2009; Gadhe et al., 2011). The predicted DFT based charges could also be useful to incorporate in docking calculations to perform virtual screening (Cho et al., 2005).

## Data Availability

The raw data supporting the conclusions of this article will be made available by the authors upon email request.
