# Supplementary material for: Quantum-based machine learning and AI models to generate force field parameters for drug-like small molecules
Source: Front Mol Biosci. 2022 Oct 11;9:1002535. doi: 10.3389/fmolb.2022.1002535 (PMC9592901; doi:10.3389/fmolb.2022.1002535)
Supplement: Supplementary file 1 [file Table1.docx]

**FIGURE S1.** Architecture of neural network models and number of neurons in each layer.

| **Model** | **Mean Square Error** |
| --- | --- |
| Random Forest Regressor | 0.0079 |
| Multi-Layer Perceptron Regressor | 0.0090 |

**TABLE S1.** Mean square error of partial charge models.

**FIGURE S2.** Partial charge prediction for (a) Sulphur (b) Fluorine (c) Chlorine (d) Bromine and (d) Phosphorous in the data set.

**TABLE S2.** Regression coefficient and MSE for each atom in the data set with respect to their DFT charges.

| Atom | R^2^ | MSE |
| --- | --- | --- |
| Carbon | 0.871 | 0.0148 |
| Hydrogen | 0.847 | 0.001 |
| Oxygen | 0.852 | 0.002 |
| Nitrogen | 0.880 | 0.013 |
| Sulphur | 0.977 | 0.004 |
| Fluorine | 0.632 | 0.0003 |
| Chlorine | 0.805 | 0.004 |
| Bromine | 0.714 | 0.001 |
| Phosphorous | 0.664 | 0.027 |

**TABLE S3.** The comparison of atomic charges for aniline molecule using ATB, random forest regression, DFT and AM1-BCC methods.

| Atoms | ATB | Random Forest | DFT | AM1-BCC |
| --- | --- | --- | --- | --- |
| N | 0.399 | 0.40767 | 0.33616 | 0.3868 |
| C | -0.969 | -0.89627 | -0.78185 | -0.8182 |
| C | 0.399 | 0.4171 | 0.33612 | 0.3868 |
| C | 0.543 | 0.15028 | 0.354435 | 0.1366 |
| C | -0.351 | -0.33814 | -0.25917 | -0.191 |
| C | 0.168 | 0.17402 | 0.141748 | 0.13 |
| C | -0.071 | -0.12173 | -0.0987 | -0.093 |
| H | 0.123 | 0.13258 | 0.116694 | 0.129 |
| H | -0.237 | -0.10402 | -0.16107 | -0.173 |
| H | 0.127 | 0.1404 | 0.11508 | 0.131 |
| H | -0.071 | -0.09552 | -0.09822 | -0.093 |
| H | 0.123 | 0.15791 | 0.116525 | 0.129 |
| H | -0.351 | -0.18217 | -0.2598 | -0.191 |
| H | 0.168 | 0.15784 | 0.142046 | 0.13 |

**TABLE S4.** Comparison of predicted partial charges from random forest model with DFT charges.

**TABLE S5.** Comparison of predicted partial charges from random forest model with DFT charges in the case of 1-Octanol.

| Atom | Random Forest | DFT |
| --- | --- | --- |
| C1 | 0.26087 | 0.280054 |
| C2 | -0.11283 | -0.073139 |
| C3 | 0.08411 | -0.019678 |
| C4 | 0.15240 | 0.165653 |
| C5 | 0.10155 | -0.084982 |
| C6 | 0.09517 | -0.020610 |
| C7 | 0.08756 | 0.177902 |
| C8 | -0.33590 | -0.301491 |
| O | -0.73002 | -0.624799 |
| H | 0.34528 | 0.383656 |

Test set-1 Test set-2


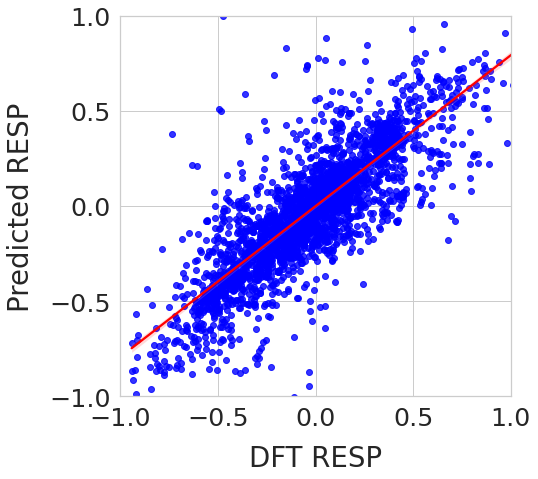

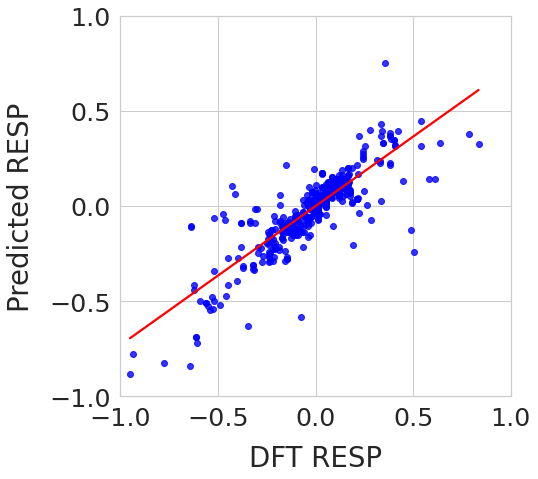


R^2^=0.61

R^2^=0.71

**FIGURE S3**. The prediction of RESP charges for the molecules in testset-1 and testset-2.

**TABLE S6.** Comparison of predicted atom types from neural network model with antechamber generated atom types.

**TABLE S7.** Comparison of predicted phase angles and periodicity for phenol and 2-methylphenol molecules with antechamber generated values.

phenol

2-methyl phenol

**TABLE S8.** Calculated solvation free energies using AI force field compared with experimental free energy values, AM1-BCC and RESP. For few molecules values are not available in the case of RESP.

| Molecule | Machine learning  (kcal/mol) | AM1-BCC(kcal/mol) | Experimental  (kcal/mol) | RESP  (kcal/mol) |
| --- | --- | --- | --- | --- |
| chloroethane | -0.012 | -0.63 | -0.39 |  |
| Ethylamine | -3.97 | -3.04 | -4.5 | -3.51 |
| 1-Octanol | -4 | -2.49 | -4.09 | -4.53 |
| Cyclopentanol | -4.7 | -4.03 | -5.49 | -6.35 |
| Benzaldehyde | -4.38 | -4.44 | -4.02 | -3.5 |
| Pentanal | -4.59 | -1.94 | -4.47 | -3 |
| Acetamide | -9.1 | -8.43 | -9.71 | -9.15 |
| aniline | -5.38 | -5.94 | -5.5 | -6.74 |
| Anthracene | -2.9 | -3.93 | -3.95 | -3.23 |
| bromobenzene | -1.51 | -0.27 | -1.46 | -0.28 |
| Cyclohexane | 1.55 | 1.91 | 1.23 | 1.4 |
| dimethylacetamide | -9.2 | -7.29 | -8.5 | -6.53 |
| Ethanediol | -7.98 | -10.16 | -9.3 | -12.89 |
| Hexanol | -3.54 | -2.88 | -4.36 | -4.27 |
| nitrobenzene | -4 | -3.18 | -4.12 | -4.9 |
| piperazine | -7.1 | -7.94 | -7.4 | -7.09 |
| Toluene | -0.91 | 0.26 | -0.89 | -0.26 |
| tricholorodibenzo-P-dioxin | -3.87 | -3.2 | -4.05 |  |
| trifluroethnol | -3 | -4.4 | -4.31 | -4.53 |
| biphenyl | -1.76 | -2.68 | -2.23 | -2.55 |
| dibenzo-P-dioxin | -2.04 | -3.98 | -3.15 |  |
| 1,2,3,4-tetrachloro-5-phenyl-benzene | -3.56 | -2.35 | -3.48 |  |
| 1,2,3,4,6,7,8,9-octachlorodibenzo-p-dioxin | -4.14 | -1.79 | -4.53 |  |

| Molecule | Charge Correction  free energy  (kcal/mol) | AM1-BCC/GAFF  (kcal/mol) | Experimental  Free Energy  (kcal/mol) | RESP/GAFF  (kcal/mol) |
| --- | --- | --- | --- | --- |
| 1-phenylethanone | -4.9 | -4.58 | -4.58 | -4.33 |
| cyclopentanone | -4.54 | -3.84 | -4.68 | -4.52 |
| chlorobenzene | -0.22 | -0.04 | -1.12 | -0.17 |
| ethanethiol | -1.34 | 0.09 | -1.34 | 0.27 |
| 2-methylphenol | -6.19 | -5.61 | -5.87 | -5 |
| Acetonitrile | -3.58 | -1.72 | -3.89 | -2.92 |
| benzenethiol | -2.2 | -1.69 | -2.55 | -0.76 |
| benzonitrile | -3.46 | -2.76 | -4.1 | -3.86 |
| methoxybenzene | -3.74 | -1.67 | -3.73 | -2.35 |
| pyridine | -4.63 | -3.16 | -4.74 | -2.82 |

**TABLE S9**. Charge correction for atoms in involved in specific bonds.

| **Molecule** | **Aromatic bonds** | **Charge correction** |
| --- | --- | --- |
| pyridine | ca-nb | 0.07 |
| nitrobenzene | ca-no | -0.08 |
| aniline | ca-nh | 0.06 |
| fluorobenzene | ca-f | 0.13 |
| chlorobenzene | ca-cl | 0.15 |
| bromobenzene | ca-br | 0.19 |
| benzonitrile | cg-n1 | 0.09 |
| phenol | ca-oh | 0.39 (only for ca) |
|  | **Aliphatic and cyclic bonds** |  |
| dimethyl sulfoxide | o-s4 | 0.06 |
| cyclohexanol | c3-oh | 0.10 |
| cyclohexanone | c-o | 0.05 |
| dimethyl sulfide | c3-ss | 0.08 |
| ethanethiol | c3-sh | 0.175 |
| nitroethane | no-o | -0.18 |
| ethyl chloride | c3-cl | 0.10 |
| ethyl bromide | c3-br | 0.08 |
| ethylamine | c3-n3 | 0.04 |
| pyrrolidine | c3-n3 | 0.09 ( only for n3) |
| acetonitrile | c1-n1 | 0.03 |
| acetamide | c-c3 | 0.3 |
| acetamide | c-n | -0.08 |
| dimethylacetamide | n-c3 | -0.18 for n, 0.1 for c3 |

| A   | B   |
| --- | --- |
| C   | |

**FIGURE S4**. (A) interaction energy between protein and ligand (B) electrostatic contribution to interaction energy (C) van der Waals contribution to interaction energy.

**Prediction of force constant for bonds, angles and dihedral angles**

Stretching of bonds, bending of angles and rotation of torsion angles depends on the strength of bonds involved those actions. These parameters are important for energy of a molecule on the potential energy surface. The derivation of these parameters from experiments and quantum mechanical calculations is difficult for small molecules. The force constants values for small molecules in GAFF are determined using approximations and which gives accurate results for bonds, angles, proper and improper dihedrals. Force field parameters for 31770 molecules have generated using antechamber programme in GROMACS format. From these topologies, bond, angle and dihedral terms extracted individually along with force constant values using our own written scripts. The atom indexes in these terms replaced with respective atomic numbers of atoms for all 31770 molecules. The atom features such as aromatic or not, present in ring or not, having double bond, hybridization and present in fused ring or not, which can describe nature of atom are included in data of bonds, angles and dihedral terms.

Training has been performed individually for force constants of bonds, angles and dihedral angles. For bonds, 13 features such as atomic numbers of atoms involved in a bond and bond length, aromatic nature of atoms, present in ring or not, hybridization, having double bond or not and present in fused ring or not are used to do train the model in order to predict force constant. We have chosen deep neural network sequential model with 1 input layer, 2 hidden layers and 1 output layer. Similarly with atomic numbers of three atoms involved in angle and their atom features, atomic numbers of four atoms involved in dihedral angle and their atom features in the training angle and dihedral angle force constants, respectively. In all the cases, relu activation function and learning rate is 0.001 was used with 500 iterations and other parameters was kept as default. As it is prediction of numbers, mean square error is used for loss function and evaluation of model. Mean square error for bonds, angle and dihedral force constants are 0.15, 0.13 and 0.11 respectively. These models predict force constants by identify atomic number and its nature, bond lengths and angle values. The predicted values are within the range and not deviated much when compared to antechamber assigned values.

The trained models are tested on randomly selected molecules and compared with GAFF parameters. The force constant values are not exactly similar to GAFF parameters. The predicted force constant values are used in molecular dynamics simulations of few small molecules. It does not induce any structural distortions in molecules and behavior of molecule.
